# The knockdown of H19lncRNA reveals its regulatory role in pluripotency and tumorigenesis of human embryonic carcinoma cells

**DOI:** 10.18632/oncotarget.5787

**Published:** 2015-09-22

**Authors:** Evelyne Zeira, Rinat Abramovitch, Karen Meir, Sharona Even Ram, Yaniv Gil, Baruch Bulvik, Zohar Bromberg, Or Levkovitch, Nathalie Nahmansson, Revital Adar, Benjamin Reubinoff, Eithan Galun, Michal Gropp

**Affiliations:** ^1^ The Goldyne Savad Institute of Gene Therapy, Hadassah Hebrew University Medical Center, Jerusalem, Israel; ^2^ The Department of Pathology, Hadassah University Hospital, Jerusalem, Israel; ^3^ The Sydney and Judy Swartz Human Embryonic Stem Cell Research Center, Hadassah-Hebrew University Medical Center, Jerusalem, Israel

**Keywords:** H19lncRNA, oncogenesis, pluripotency, hEC cells

## Abstract

The function of imprinted H19 long non-coding RNA is still controversial. It is highly expressed in early embryogenesis and decreases after birth and re-expressed in cancer. To study the role of H19 in oncogenesis and pluripotency, we down-regulated H19 expression *in vitro* and *in vivo* in pluripotent human embryonic carcinoma (hEC) and embryonic stem (hES) cells. H19 knockdown resulted in a decrease in the expression of the pluripotency markers Oct4, Nanog, TRA-1-60 and TRA-1-81, and in the up-regulation of SSEA1; it further attenuated cell proliferation, decreased cell-matrix attachment, and up-regulated E-Cadherin expression. SCID-Beige mice transplanted with H19 down-regulated hEC cells exhibited slower kinetics of tumor formation, resulting in an increased animal survival. Tumors derived from H19 down-regulated cells showed a decrease in the expression of pluripotency markers and up-regulation of SSEA-1 and E-cadherin. Our results suggest that H19 oncogenicity in hEC cells is mediated through the regulation of the pluripotency state.

## INTRODUCTION

The imprinted H19 gene encoding a long non-coding RNA (lncRNA) transcript of 2.3 kb, is part of the igf2/H19 imprinted gene cluster located on chromosome 11p15.5 in humans [1]. In addition to the H19 lncRNA, miR-675, a highly conserved microRNA involved in the regulation of developmental genes, is expressed from the H19 gene [2]. H19 lncRNA is highly expressed in the placenta and embryonic tissues and is strongly repressed shortly after birth in most tissues, except for the cardiac muscle, skeletal muscle, and cartilage [3]. However, it is re-expressed in several cancers [4-5]. Conflicting data suggest that H19 functions as a tumor suppressor [6] as well as an oncogene [4].

Previous studies have demonstrated that lncRNAs play a role in the maintenance of pluripotency in mouse embryonic stem (mES) cells and several lncRNAs were further shown to be regulated by the key pluripotency transcription factors Oct4, Sox2 and Nanog [7-8]. Several reports have indicated a possible link between H19 lncRNA and pluripotency: H19 transcripts were detected in mouse and human embryos early at the pre-implantation stage [9], and monoallelic-maternal H19 expression was observed in human pluripotent ES (hES) cells [10]. Moreover, in mES cells, Oct4 and Sox2 were shown to cooperatively bind the imprinting control region of the Igf2/H19 locus, establishing its hypomethylated active state [11].

The aim of the current study was to determine whether H19 regulates pluripotency and tumorigenicity. Two experimental cell types were used: (1) a human embryonic stem (hES) cell line HES-1 [12] and (2) two human embryonic carcinoma (hEC) cell lines - NCCIT [13] and Ntera2 (NT2) [14]. hES cells are pluripotent cells derived from preimplantation embryos. These cells can self-renew for long periods, and have the potential to differentiate into any cell type [12]. hEC cells are derived from teratocarcinomas, germ cell tumors, and have both pluripotent and tumorigenic characteristics [15]. hEC cells were shown to constitute an alternative model system to investigate pluripotency and differentiation in human embryonic research [16-17]. Similarly to ES cells, hEC cells are capable of unlimited self-renewal and can differentiate into all three germ layers [18]. Both NCCIT and NT2 cells express the key pluripotency transcription factors Oct4, Sox2, and Nanog. In addition, NT2 and NCCIT cells have several advantages: they grow without feeder cells, are relatively simple to passage and resist spontaneous differentiation [19].

Here we show that the inducible knockdown of the H19 gene in hES and hEC cells down-regulated the expression of the key pluripotency transcription factors Oct4 and Nanog and promoted the expression of early differentiation markers SSEA1 and Nestin. H19 knockdown attenuated hEC cell proliferation and induced cell detachment, changes in the cytoskeleton and up-regulation of E-Cadherin. These *in vitro* results were further confirmed by *in vivo* experiments. Our data highlights the involvement of H19 in regulating the pluripotency of human EC and ES cells suggesting its role in tumorigenesis.

## RESULTS

### Human ES and EC cells express H19 and pluripotency markers

Prior to studying the involvement of H19 in pluripotency, we analyzed by RT-PCR the basal expression levels of H19, and the key pluripotency transcription factors OCT4, Nanog and Sox2 in NCCIT, NT2 and HES-1 cells. All three cell lines expressed H19 as well as the three pluripotency factors (Supplementary Figure.S1A). We further assessed the surface antigen expression of the pluripotency-associated markers Tra-1-60 and Tra-1-81 by flow-cytometry. The majority of both NCCIT and HES-1 cells expressed TRA-1-60 and TRA-1-81 (Supplementary Figure.S1B).

### A regulated system for the inducible knockdown of the H19 gene in hES and hEC cells

A tetracyclin (tet)-inducible lentiviral-RNAi system was used to target H19 in hES and hEC cells. In order to determine the siRNA that could be used for efficient down-regulation of H19, NCCIT cells were transiently transfected with two H19 siRNAs: siRNA_1_ and siRNA_3_ [4] and two control siRNAs: Luc siRNA and Scramble siRNA (Supplementary Table S1). RT-PCR and real-time PCR (qPCR) showed efficient knockdown of H19 by both synthetic H19siRNAs compared to the two siRNA controls (Supplementary Figure.S2A and S2B). Therefore we chose the Luc siRNA and H19 siRNA_1_ for constructing the inducible knockdown of the H19 gene.

To study the loss of function of H19, we transduced human ES and EC cells with lentiviral vectors, harboring a tet-inducible H19-shRNA or a control luciferase (Luc)-shRNA, and a constitutive tet-repressor fused to GFP (a description of the vectors is found in Supplementary Information and Supplementary Figure.S2C and S2D). Transductions were highly efficient, resulting in the majority of cells expressing GFP during long culturing periods (Supplementary Figure.S3A). In the absence of Doxycycline (Dox), the transduction did not affect cell morphology, and the percentages of cells expressing TRA-1-60 and TRA-1-81 were only slightly reduced (Supplementary Figure.S3B).

**Figure 1:**
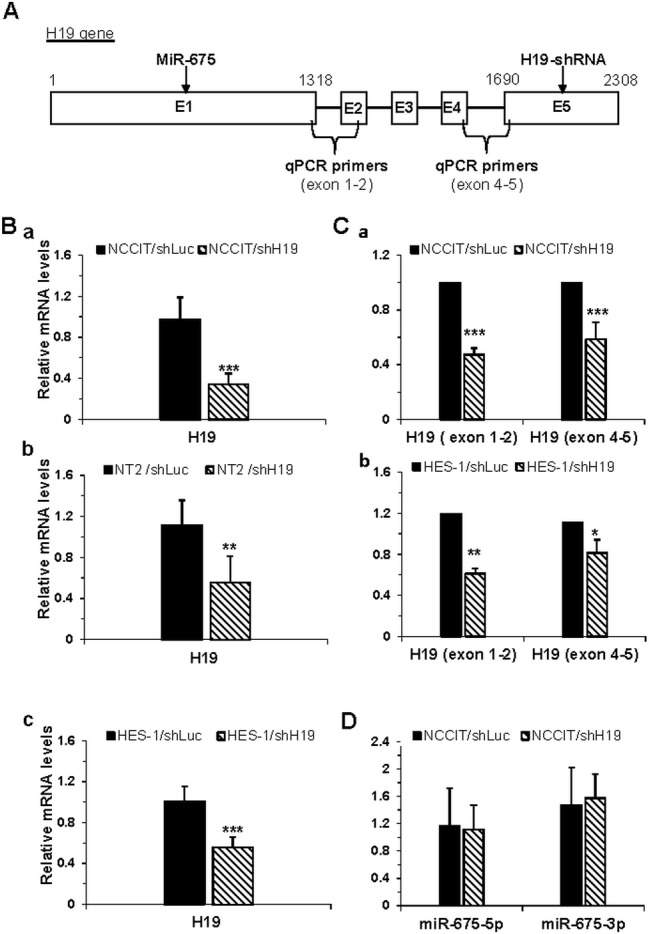
Efficient inducible knockdown of the H19 gene in transduced hES and hEC cells **A.** Schematic representation of the human H19 gene. The genomic site of miR-675, the target site of H19-shRNA and the primer sites used in qPCR assays are marked. **B.** The relative expression levels of the H19 mRNA as assessed by qPCR, reveals efficient down-regulation of H19 in NCCIT cells (a; *n* = 14), NT2/shH19 cells (b; *n* = 3) and HES-1 cells (c; *n* = 5) compared to controls transduced with shLuc. **C.** The entire H19 mRNA was down-regulated as assessed by qPCR using primers over the first intron (exon 1-2) for NCCIT cells (a, *n* = 2) and for HES-1 cells (b, *n* = 2). **D.** miR-675 expression in NCCIT cells was stable, and was not affected by H19 down-regulation as detected by qPCR (*n* = 3). All the expression levels are normalized to the housekeeping gene β-actin. Data are represented as mean ± SD; *-*p* ≤ 0.05, **p ≤ 0.01, ***p ≤ 0.001.

The addition of Dox to the growth medium of shH19-transduced cells for three days induced a significant down-regulation of H19 expression levels compared to control cells as measured by qPCR using primers designed to span exons 4 and 5 (Figure.1A and Ba-Bc). Since the H19-shRNA targets exon 5 of the H19 gene (Figure.1A), we further verified that the inhibition affected the entire H19 gene by using additional primers spanning exons 1 and 2 of the gene. A comparable inhibition of H19 gene expression was measured with both primer sets (Figure.1C a-b). Therefore all experiments were performed three days after induction of Dox unless stated otherwise.

Next, we examined whether the inhibition of H19 gene expression affected the expression of miR-675, located at exon 1 of the gene (Figure.1A). Notably, knockdown of H19 gene expression in NCCIT cells had no effect on the expression of miR-675 relative to the control NCCIT cells (Figure.1D), confirming previous data showing that miR initial processing is carried out in the nucleus while the processing of shRNA into functional siRNA is performed in the cytoplasm [20-21]. Thus the observed effects of H19 knockdown could be attributed exclusively to H19 lncRNA.

### H19 knockdown decreases the pluripotency of human stem cells and promotes early differentiation

**Figure 2:**
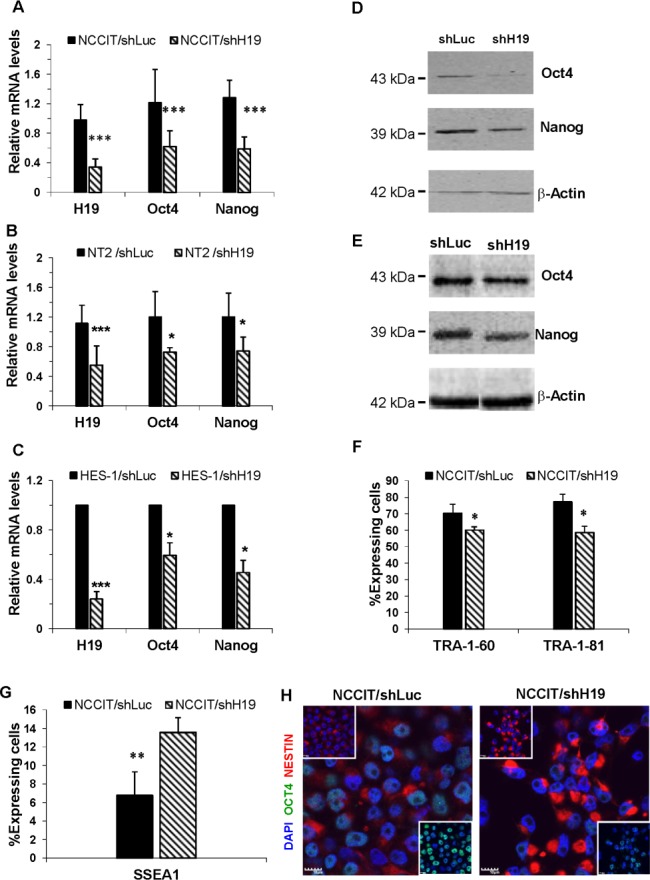
H19 knockdown decreases pluripotency of hES and hEC and promotes early differentiation **A.-E.** Down-regulation of H19 expression decreases the RNA and protein expression levels of Oct4 and Nanog in hES and hEC cell lines. **A.** qPCR of RNA from NCCIT cells for Oct4 (*n* = 10) and Nanog (*n* = 9). **B.** qPCR of RNA from NT2 cells for Oct4 (*n* = 3) and Nanog (n = 3). **C.** qPCR of RNA from HES-1 cells for Oct4 (*n* = 2) and Nanog (*n* = 2). **D.** Western blot of Protein lysates from NCCIT cells for Oct4 and Nanog **E.** Western blot of Protein lysates from NT2 cells for Oct4 and Nanog **F.** H19 knockdown in NCCIT cells reduces the expression levels of surface markers TRA-1-60 and TRA-1-81, as analyzed by FACS (*n* = 2).**G.** H19 down-regulation induces an increase of SSEA1 expression in shH19 transduced NCCIT cells, as analyzed by FACS (*n* = 4).**H.** H19 down-regulation in transduced NCCIT cells induces a decrease of Oct4 (green) and an increase of Nestin (Red) expression, as assessed by immunofluorescence (Nuclear counterstaining with Dapi (Blue)). Data are represented as mean ± SD; *p ≤ 0.05, **p ≤ 0.01, ***p ≤ 0.001.

Down-regulation of H19 expression for three days caused a significant decrease in the RNA and protein expression levels of the pluripotency transcription factors Oct4 and Nanog in all three transduced cell lines (Figure.2A-2E). On the other hand, no detectable changes were observed for the transcription factor Sox2 (data not shown). Since all three cell lines (hEC and hES cells) showed similar results, we focused our study on the NCCIT cell line.

Silencing of H19 expression for a longer period (eight days) in NCCIT cells was followed by a significant reduction of the pluripotency surface markers TRA-1-60 and TRA-1-81 (Figure.2F), and up-regulation of the stage-specific embryonic antigen 1 (SSEA1), a marker of differentiation, which is not expressed by undifferentiated human EC and ES cells [22] (Figure.2G).

To further investigate the link between down-regulation of H19, pluripotency and early differentiation, transduced NCCIT cells were plated on laminin and poly-L-Lysine coated wells and then immunostained for Oct4 and Nestin, an early neural differentiation marker [23]. A significant decrease of Oct4 and a marked increase of Nestin intensities were observed in NCCIT/shH19 cells compared to control cells (Figure.2H).

These results suggest that H19 is involved in the regulation of human ES and EC cells pluripotency, and that its down-regulation promotes the early differentiation of these cells.

### H19 knockdown attenuates hEC cell proliferation

**Figure 3:**
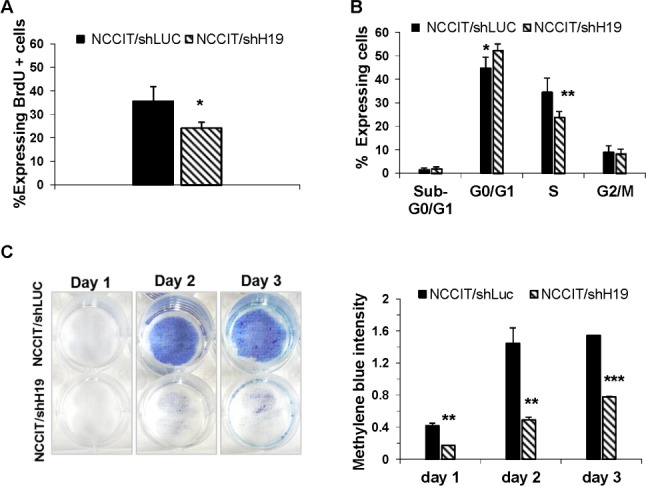
H19 inhibition attenuates hEC cell proliferation **A.** Down-regulation of H19 in NCCIT cells induces a reduction of cell proliferation as assessed by the BrdU incorporation assay (*n* = 3). **B.** FACS analysis of the cell cycle of the transduced NCCIT/shH19 cells reveals a decrease in cells residing in S phase, and a shift from S phase to G0/G1 phase (*n* = 3). **C.** A decrease in cell numbers following H19 down-regulation in NCCIT/shH19 as revealed by Methylene blue assay. Data are represented as mean ± SD; *p ≤ 0.05, **p ≤ 0.01, ***p ≤ 0.001.

Pluripotent stem cells are defined by their ability to self-renew, and are characterized by rapid cell cycling, with shortened G1-phase and high percentages of cells residing at the S-phase [24-25]. Therefore we investigated whether H19 inhibition would affect human EC cell proliferation. Down-regulation of H19 in NCCIT cells induced a marked reduction of cell proliferation as confirmed by the BrdU incorporation assay (Figure.3A). Analysis of the cell cycle status of the transduced cells, revealed that down-regulation of H19 expression led to a significant decrease in cells residing in S phase, and a shift from S phase to G0/G1 phase (Figure.3B). The reduced cell proliferation resulted in prolonged doubling times (data not shown). Methylene Blue assay in transduced NCCIT/shH19 cells revealed a decrease in viable cell numbers compared to control cells (Figure.3C). Apoptosis was not detected in the transduced cells using the AnnexinV apoptosis detection Kit (data not shown). This was confirmed by the low levels of pre-apoptotic cells residing in the sub G0/G1 phase in both control and H19 down-regulated NCCIT cells (Figure.3B). These results suggested that H19 supports human EC cell proliferation.

### H19 knockdown affects hEC cell-matrix and cell-cell interactions

**Figure 4:**
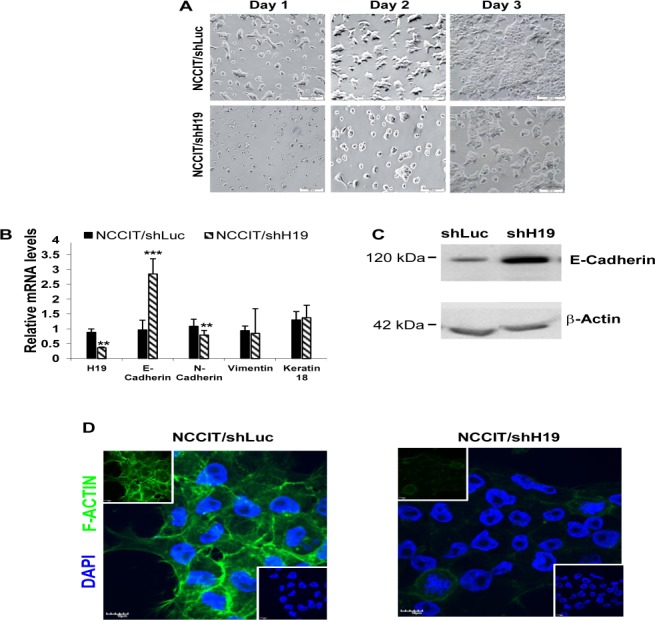
H19 knockdown decreases hEC cell-matrix attachment and decreases hEC cell-cell interactions **A.** Down-regulation of H19 expression in NCCIT cells causes a marked change in cell morphology with failure of the cells to attach to the matrix. **B.** H19 inhibition in NCCIT induces up-regulation of E-Cadherin and down-regulation of N-Cadherin expression while Vimentin and Keratin-18 expression levels remain unchanged (qPCR; *n* = 4). **C.** Up-regulation of E-Cadherin protein levels in NCCIT/shH19 cells as detected by Western blot. **D.** NCCIT/shH19 cells exhibit a decreased organization of F-actin: Immunohistofluorescence staining for F-Actin (Green) and nuclear counterstaining with Dapi (Blue).

The down-regulation of H19 expression in NCCIT cells caused a marked change in cell morphology. During the first days of culture, we observed clusters of flattened cells as well as floating viable cells (Figure.4A). The methylene blue assay further indicated a failure of the cells to attach to the matrix (Figure.3C). Cells often convert cell-matrix to cell-cell adhesions in attempt to maintain adhesion-dependent survival signals. We therefore assessed the expression level of E-Cadherin, an important regulator of cell-cell adhesion and tissue morphology [26]. qPCR analysis showed a lower expression level of E-Cadherin in non-transduced NCCIT cells compared to HES-1 cells (data not shown). Upon inhibition of H19 in NCCIT cells a significant increase of E-Cadherin expression was demonstrated by qPCR (Figure.4B) and Western-blot analysis (Figure.4C). To study whether the up-regulation of E-Cadherin in NCCIT/shH19 is associated with epithelial-mesenchymal transition (EMT), we analyzed the expression levels of additional EMT markers. qPCR showed a reduction in N-Cadherin, while Vimentin and Keratin-18 expression levels remained unchanged (Figure.4B). Down-regulation of H19 in transduced NCCIT cells was also followed by a decreased organization of stress Actin fibers (F-Actin) compared to the control (Figure.4D).

These results suggested an involvement of H19 in the regulation of cell-cell and cell-matrix interactions as well as cytoskeleton organization in pluripotent cells.

### Xenografts from NCCIT/shH19 cells show down-regulation of pluripotency and up-regulation of early differentiation markers

**Figure 5:**
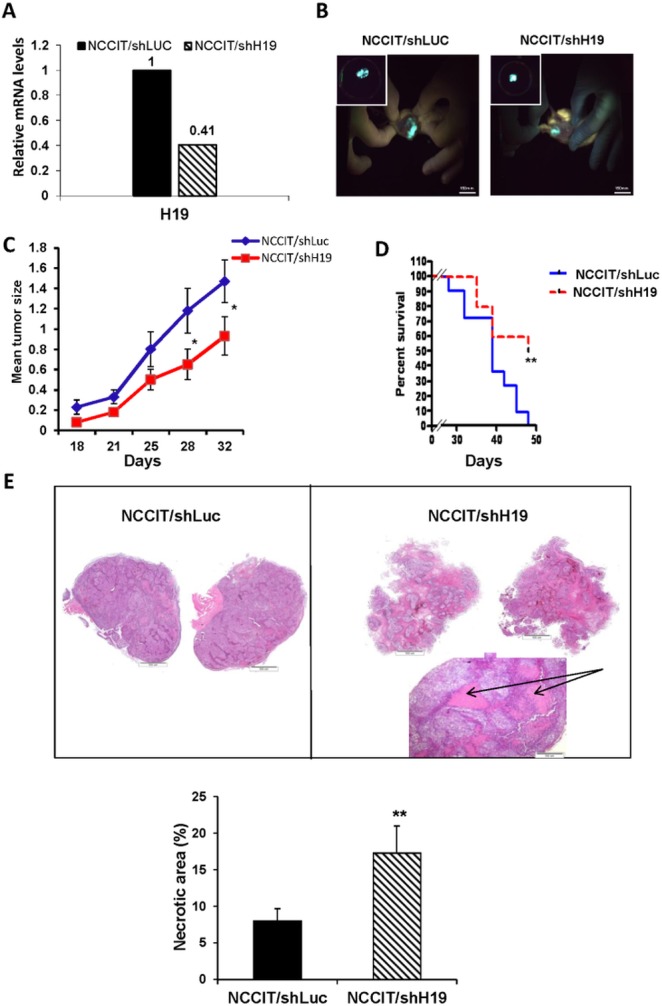
Xenografts from NCCIT/shH19 cells show attenuated tumor growth and massive necrosis **A.** qPCR confirming the efficient knockdown of H19 gene expression prior to transplantation. **B.** Tumors derived from transduced hEC cells express GFP. **C.** Tumor growth monitoring up to day 32 after transplantation of NCCIT/shLuc (*n* = 11) and NCCIT/shH19 (*n* = 10) cells shows that H19 silencing attenuates tumor growth. Data are represented as mean ± SEM; *p ≤ 0.05. **D.** Kaplan-Meier plot representing the percentages of survival shows prolonged survival of mice transplanted with NCCIT/shH19 cells (**p ≤ 0.01). **E.** H&E staining of representative tumor sections derived from NCCIT/shLuc (left) and NCCIT/shH19 (right) demonstrating enhanced necrosis in the NCCIT/shH19 tumors (upper panel). Quantitation of the necrotic areas in the tumors showing higher percentages of necrotic areas in NCCIT/shH19 derived tumors compared to NCCIT/shLuc derived tumors (**p ≤ 0.01) (Lower panel).

**Figure 6:**
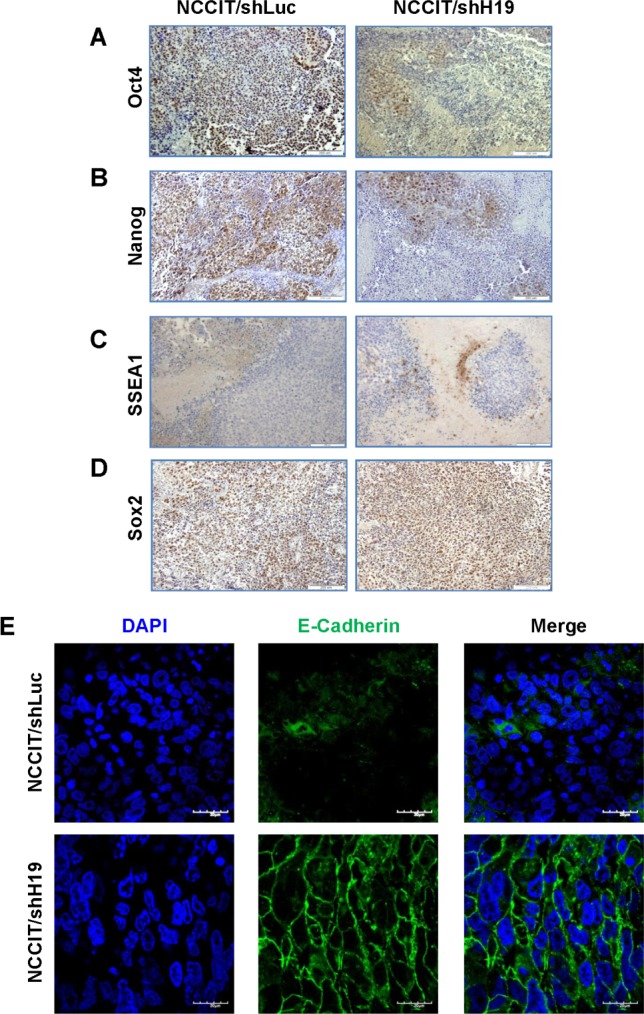
NCCIT/shH19 derived xenografts show down-regulation of Oct4 and Nanog and up-regulation of SSEA-1 and E-Cadherin **A.-D.** Immunohistochemical staining of **A.** Oct4 **B.** Nanog **C.** SSEA-1 **D.** Sox2 (Left panel NCCIT/shLuc derived tumors, right panel- NCCIT/shH19 derived tumors). **E.** Immunofluoresence staining of E-Cadherin.

The human EC cell line, NCCIT, has the capacity to produce complex xenograft tumors and to give rise to the differentiated tissues within teratocarcinoma. Since our *in vitro* studies suggested that H19 plays a regulatory role in pluripotency and early differentiation, we investigated the effect *in vivo* of H19 silencing in teratocarcinomas derived from transduced NCCIT cells. Prior to transplantation, the efficiency of H19 knockdown in NCCIT cells was confirmed (Figure.5A). The transduced cells were implanted subcutaneously into severe combined immune-deficient (SCID)/Beige mice and tumor growth was monitored until tumors reached ethical restrictions or for a period of 50 days. The tumors expressed GFP, indicating the presence of transduced cells (Figure.5B). Delayed tumor formation and a decrease in tumor size were observed in tumors derived from down-regulated H19 cells compared to shLuc transduced cells (Figure.5C). Moreover, a Kaplan-Meier analysis showing the percentages of surviving transplanted mice at various time-points, revealed that H19 silencing prolonged mouse survival significantly (*p* < 0.01; Figure. 5D). H&E staining of NCCIT/shH19 tumors demonstrated that H19 down-regulation induced massive necrosis which was further quantified demonstrating significantly higher percentages of necrotic areas compared to the control tumors (Figure.5E). Immunohistological analysis revealed reduced expression levels of Oct4 and Nanog in the NCCIT/shH19 derived tumors (Figure.6A and 6B respectively). On the other hand, increased staining of SSEA1 was detected in the NCCIT/shH19 derived tumors. Notably most of the SSEA1 staining was detected at the periphery of the necrotic foci (Figure.6C). Additionally, a stronger membranal staining of E-cadherin was evident in the NCCIT/shH19 derived tumors compared to control (Figure.6E). In contrast to the observed effects of H19 down- regulation on Oct4, Nanog, SSEA1 and E-cadherin, the levels of Sox2 remained high in controls and shH19 tumors (Figure.6D).

These *in vivo* results are in accord with our *in vitro* observations, thus supporting a possible role of H19 in the regulation of pluripotency and early differentiation. They further suggest a role for H19 in tumorigenesis.

## DISCUSSION

The imprinted H19 gene encodes a lncRNA implicated as having both oncogenic and tumor suppression properties. In this study, we aimed to investigate the role of H19 in pluripotency and tumorigenesis. Using an inducible lentiviral-based system, we targeted H19 in hES (HES-1) and hEC (NCCIT and NT2) pluripotent cells. Although NCCIT and NT2 cells are derived from teratocarcinoma tumors, they share many properties with hES cells. They encounter the capacity to self-renewal as well as differentiation, the expression of the core pluripotency transcriptional regulators Oct4, Nanog, and Sox2 [27], the expression of the surface antigens TRA-1-60 and TRA-1-81, and lack of expression of SSEA1 [22-28].

Induction of shH19 expression in transduced cells resulted in a significant down-regulation of H19 mRNA expression. Notably, H19 knockdown had no effect on mir-675 expression, indicating that the observed effects of H19 knockdown could be attributed exclusively to H19 lncRNA.

Down-regulation of H19 lncRNA in hES and hEC cells induced a decrease in the expression levels of Oct4 and Nanog. These results are supported by previous studies showing that knockdown of mES-specific lncRNAs decreased significantly both Nanog and Oct4 expression levels, causing an exit from the pluripotent state [7-8]. A decrease in the expression of the pluripotency transcription factors Oct4 and Sox2 was also observed in human prostate cancer cells following knockdown of H19 [29]. Together with the decrease in Oct4 and Nanog expression, we observed a down-regulation of the surface antigen markers TRA-1-60 and TRA-1-81 and induction of SSEA1, a marker associated with early differentiation of hES/hEC cells that was shown to be strongly expressed by human trophectoderm [22, 30-31]. H19 silencing further induced an increase in the protein levels of nestin, an intermediate filament protein marker of neural stem cells expressed during the early stages of development in the central nervous system [32]. Recently, H19 lncRNA was shown to prevent precocious muscle differentiation by inhibiting let-7 miR activity [33].

Undifferentiated hES and hEC cells can self-renew in culture for long periods. Their rapid cell-cycle is characterized by shortened G1-phase and high percentages of cells residing at the S-phase [24, 34]. The cell-cycle machinery of ES cells was shown to be involved in the maintenance of pluripotency [25]. Knockdown of H19 in hEC cells attenuated their proliferation and promoted a shift of cells from the S-phase to the G0/G1 phase. Since the pluripotency regulators Oct4 and Nanog were implicated in regulating ES cell**-**cycle [35], we hypothesize that the decrease of these transcription factors, following H19 down -regulation may be directly linked to the reduced proliferation. Furthermore, the higher percentages of cells residing in G0/G1 could be prone to differentiation as was suggested by Neganova and Lako [35].

H19 silencing in hEC cells induced significant changes in cell morphology with a decrease in the ability of the cells to attach to the matrix, up-regulation of E-Cadherin expression, and reorganization of the F-Actin. E-Cadherin is an important cell-cell mediator and regulator of tissue morphology [26] implicated in the regulation of pluripotency [36]. As reported earlier [15], we also observed that hEC (NCCIT) cells express low levels of E-Cadherin. Loss of function of E-Cadherin occurring in many epithelial tumors was shown to elicit invasive tumor phenotype associated with EMT [37-38]. In our study, up-regulation of E-Cadherin in hEC/shH19 cells was followed by a decrease of N-Cadherin whereas Vimentin and Keratin-18 expression levels remained unchanged, indicating that, in this case, E-Cadherin may not be correlated with EMT. Our results suggest that the increased levels of E-Cadherin may be linked to the reduced levels of Nanog, since Nanog was shown to promote invasiveness and migration of human carcinoma cancer cells via dysregulation of E-cadherin [39-40]. Furthermore, the up-regulation of E-Cadherin was found to be correlated with epithelial differentiation.

Together these *in vitro* results propose a role for H19 in the regulation of hEC/hES cell fate. They further suggest that H19 is involved in controlling hEC cell proliferation, cell-cell and cell- matrix interactions.

In accord with our *in vitro* results, NCCIT/shH19 derived teratocarcinoma tumors exhibited a delayed tumor formation indicating slower growth kinetics. Our results are supported by studies reporting that the increase of H19 expression in cancer cells contributed to the proliferation of cancer [41-42]. The shH19 derived tumors showed also massive necrotic foci suggesting non-apoptotic cell death. They also displayed reduced expression levels of Oct4 and Nanog and increased expression levels of SSEA1, confirming the study of Ben-Porath *et al.*, reporting that poorly differentiated human tumors overexpressed the pluripotency regulators Oct4, Nanog and sox2, thus linking pluripotency with tumor phenotype [43]. Furthermore, the up-regulation of E-Cadherin in NCCIT|/shH19 derived tumors may be associated with differentiation, as E-Cadherin expression was detected in differentiated carcinoma cancers [44- 45].

Our *in vivo* results suggest that H19lncRNA may play a role in hEC tumorigenesis by supporting tumor cell proliferation, decreasing cell-cell interactions, inducing pluripotency regulators and preventing differentiation. H19 and the core stemness transcription factors were shown to be overexpressed in several other cancers such as Bladder, Lung, Liver, Breast, Ovary and Prostate, [5, 46]. We further propose that H19 regulates pluripotency and oncogenesis through the regulation of Nanog and Oct4.

## MATERIALS AND METHODS

### Cell lines

Cell lines used in this study: EC cell lines NCCIT and Ntera2 (NT2), obtained from ATCC (American Type Culture Collection, Rockville, MD) and Human ES cell line HES-1 [12]. A Brief cell culture protocol is described in Supplementary Information.

### Reagents and antibodies

Gene expression was induced by adding doxycycline hydrochloride (Dox; Sigma Aldrich-Israel) to the cell culture medium (1 μg/ml), or to the drinking water (0.5 mg/ml) together with 5% Sucrose (Sigma) in the *in vivo* experiments. Commercial primary antibodies information is provided in Supplementary Information. (Supplementary Table S4)

### Inducible shRNA plasmids

ShRNAs targeting the human H19 RNA (shH19) (GenBank accession N° NR_002196) and the control firefly luciferase (from pGL3-Promega) (shLuc) were previously described [4]. Sense and antisense oligonucleotides of the shRNA duplex were synthesized by IDT Syntezza Israel and cloned into a pTER+ plasmid (modified pTER system vector [47], generously given by Prof. Hans Clevers. The inducible silencing cassette was inserted into a modified lentiviral backbone plasmid generously provided by Prof. Yinon Ben-Neriah and his team. The final modified lentiviral vector consists of an HI promoter, Tet operator, the shRNA coding sequence and EF1α promoter driving the Tet repressor fused to eGFP. Detailed siRNA duplexes (Supplementary Table S1) and vector design protocol are described in Supplementary Information and Supplementary Figure. S2.

### Lentivirus shRNA's production, transduction cells and shRNA induction

Lentiviral particle production and transduction of HES, NCCIT and NT2 cells were performed as described [48]. Brief methods are described in Supplementary Information. Positively transduced cells were fluorescence-activated cell sorted for eGFP to obtain a pure culture of transduced cells. Media with inducer (Dox) were replaced every second day for long-term knockdown experiments. Cells were harvested 3 or 8 days after induction and assayed for mRNA and protein.

### RNA extraction and reverse transcription-PCR and quantitative PCR (qPCR)

Total RNA was extracted using the Quick-RNA MiniPrep Kit (Zymo Research, CA, USA) and subsequently, cDNA was generated from 1 μg of total RNA using the Quanta Biosciences cDNA Synthesis kit. PCR amplifications were performed using gene-specific primers (Supplementary Table S2).

Primers used for qPCR validation are listed in Supplementary Table S3. A detailed method is described in the Supplementary Information.

### Western blot analysis

Cell lysates were prepared according to the Abcam Israel protocol. 30-50 μg of total protein from cell lysate were subjected to SDS-PAGE. A detailed method is described in Supplementary Information.

### Cell proliferation, cell cycle, analysis assays

Proliferation and cell cycle assays were determined by FACS analysis of BrdU incorporation and 7-amino-actinomycin D (7-AAD; eBioscience, CA, USA) using the APC BrdU Flow Kit (BD Biosciences, CA, USA). The detailed analysis method is described in Supplementary Information.

### Immunofluorescence and flow cytometry

Immunofluorescence staining was performed on paraformaldehyde-fixed cells. For the FACS analyses cells were dissociated into a single-cell suspension. In order to characterize the expression levels of pluripotency and differentiation markers, the cells were stained with specific antibodies for TRA-1-60, TRA-1-81 and SSEA1 and analyzed by flow cytometry-FACScalibur (Becton Dickinson Immunocytometry Systems), using the CellQuest software. A detailed protocol and list of antibodies and dilution ratios are available in Supplementary Information and Supplementary Table S4.

### Immunohistochemistry (IHC)

Immunohistochemical analyses were performed using standard procedures and described in Supplementary Information.

### Methylene blue dye staining

Measurement of the number of cells attached to a surface by methylene blue dye staining was carried out as described [49]

### Xenografts

Teratocarcinoma formation was carried out as described [50]. All experiments were approved by the Animal Care Committee of the Hebrew University; see detailed method in the Supplementary Information.

### Necrosis evaluation

Necrosis quantification was performed using the Ariol image analysis system (Genetix, San Jose, CA, USA) by scanning H&E-stained slides (10 and 11 lesions per group) with an automated scanning microscope.

### Statistical analyses

Data are expressed as the mean ± SD. Statistical comparisons of means were performed by a two-tailed unpaired Student's *t* test. The value of P≤0.05 was considered significant. All experiments were repeated at least three times.

## SUPPLEMENTARY MATERIAL FIGURES AND TABLES


