# Supplementary material for: The knockdown of H19lncRNA reveals its regulatory role in pluripotency and tumorigenesis of human embryonic carcinoma cells
Source: Oncotarget. 2015 Sep 22;6(33):34691–703. doi: 10.18632/oncotarget.5787 (PMC4741483; doi:10.18632/oncotarget.5787)
Supplement: Supplementary file 1 [file oncotarget-06-34691-s001.pdf]

The knockdown of H19lncRNA reveals its regulatory role in pluripotency and tumorigenesis of human embryonic carcinoma cells

Supplementary Material

Supplemental Experimental Procedures

Cell culture

EC cell lines NCCIT and Ntera2 (NT2) were obtained from ATCC (American Type Culture Collection Rockville, MD) and maintained at log growth in RPMI-1640 medium (Gibco Laboratories, New York, USA) supplemented with 10% fetal calf serum (FCS), sodium pyruvate, 1 mmol/L L-glutamine, 1% nonessential amino acids, 100 U/mL penicillin, and 0.01 mg/mL streptomycin (Biological Industries, Beit Haemek, Israel).

Human ES (hES) cells (HES-1) were cultured on mitomycin C treated human foreskin fibroblasts feeders (10 microgram/ml MMC; Sigma, St. Louis, MO, USA;) in serum free medium as described earlier (1). The cells were passaged routinely as small clusters by treatment with 1 mg/ml collagenase type IV (Invitrogen, Carlsbad, CA).

Human foreskin fibroblasts and human embryonic kidney (HEK) 293T cell lines were cultured in 90% Dulbecco's modified Eagle's medium (DMEM, Gibco-BRL, Gaithersburg, MD), supplemented with 10% fetal calf serum (FCS, Biological Industries), 1 mM L-glutamine, 100 U/ml penicillin, and 50 μg/ml streptomycin (all from Invitrogen).

Design of inducible lentiviral vectors harboring H19-specific shRNA and control Luc-shRNA

The pTER+ system (2) was generated by inserting H1 promoter into the BglII and Hind III sites of pCDNA4TO (Invitrogen). Later a 750 bp stuffer was inserted between BglII and Hind III sites (2). The sequences of shRNA targeting the H19 and the firefly luciferase (Luc) used in this study are

listed in Table S1. The sequences were generated by annealing of the sense and antisense oligonucleotide:

For the H19-shRNA:

5'GATCCCTAAGTCATTTGCACTGGTTTTCAAGAGAAACCAGTGCAAATGACTTATTTTTGGAAA 3'

5'AGCTTTTCCAAAAATAAGTCATTTGCACTGGTTTCTCTTGAAAACCAGTGCAAATGACTTAGG 3'

For the control Luc-shRNA:

5'GATCCCCTTACGCTGAGTACTTCGATTCAAGAGATCGAAGTACTCAGCGTAAGTTTTTGGAAA 3’

5’AGCTTTTCCAAAAACTTACGCTGAGTACTTCGATCTCTTGAATCGAAGTACTCAGCGTAAGGG 3'

(The H19 and Luc -siRNA sequences are underlined).

Each annealed oligonucleotide was inserted into the BglII and HindIII sites of pTER+ plasmid, containing the H1 promoter and Tetracycline Operator, to produce plasmid pTER/shLuc and pTER/shH19. The complete shH19/shLuc expression cassettes were excised from the pTERshH19/shLuc as an EcoRI fragment (Blunt ends) and inserted into the EcoRV site of a modified pRRL.sin.PPT lentiviral vector (3) containing the hEF1alpha promoter driving the expression of the tet-repressor fused to EGFP (generously given by Prof. Yinon Ben-Neriah and his team -The Lautenberg Center for Immunology, The Hebrew University, Jerusalem, Israel). The final modified lentiviral vector pRRL.sin.cPPT H1/TO-shRNA hEF1α-TetR-EGFP harbors an HI promoter, tet-operator driving the induced expression of the shRNAsequence and hEF1alpha promoter driving the expression of the tetrepressor fused to EGFP. Cloning strategy is depicted in Fig. S2.

Production of lentiviruses and transduction of hES and hEC cells

Production of lentiviral particles and transduction of HES-1, NCCIT and NT2 cells were performed as described (4, 5). Briefly, high-titer lentiviral particles (titers of $3.2x10E^8$ transduction units/ml) were generated by transfecting HEK 293T cells cultured on 10-cm plates, with 10 μg of the lentiviral plasmid encoding the silencing cassettes (shH19/shLUC), 6.5 μg of the packaging plasmid

pCMVΔR8.91, and 3.5 μg of the envelope protein (pMD.G) (a total of 20 μg plasmid DNA) as described previously (5-7). The supernatant containing the viral particles was collected, concentrated, using an ultracentrifuge (Sorvall ultracentrifuge model Discovery 100, with a Surespin 630 swinging bucket rotor) and used to transduce hES and hEC cells. Transduction was carried out as previously described, but without the presence of polybrene. (8). Briefly, prior to transduction, hES cells were dissociated into a single cell suspension with 0.05% EDTA solution (biological Industries). 1×10^5 cells were incubated with the concentrated viral supernatant and plated on feeders in a well of six-well dish. For transduction of NCCIT and NT2 lines, 2.5×10^5 cells/well of six-well dish were plated 24 hours before the transduction and then incubated with the concentrated viral supernatant.

RNA Extraction, Reverse Transcription-PCR and PCR

Total RNA was extracted using the Quick-RNA MiniPrep Kit (Zymo Research, CA, USA). Integrity of RNA was checked by agarose gel electrophoresis and ethidium bromide staining. Subsequently, cDNA was generated from 1 μg of total RNA using Quanta bioSciences cdna synthesis kit according to the manufacturer's instructions (Quanta Bioscience, Gaithersburg, USA).

miR cDNA was generated from 500ng of total RNA using Quanta Bioscience microRNA cDNA Synthesis Kit according to the manufacturer's instruction (Quanta Bioscience).

cDNA was amplified by PCR using Ready mix for PCR (Bio-Lab, Jerusalem, Israel) and specific primers (Table S2). Cycle parameters for all genes were 30 s at 94 °C, 30 s at 55-60°C, and 30 s at 72°C for 29 cycles, and a final extension of 5 min at 72°C. PCR products were separated on 1% agarose gel.

Quantitative PCR (qPCR)

qPCR reactions were performed with the 7900HT Fast Real-Time PCR System (Applied Biosystems, Carlsbad, USA) in a total reaction volume of 20 µl using 100ng cDNA for H19 expression and 30ng cDNA for the other genes, PerfeCTa® SYBR® Green FastMix® (Quanta BioSciences, Inc.), and 500 nM of forward and reverse primers. All qPCR assays were performed in triplicates and the relative gene expression was determined after normalization with beta-Actin or GUSB transcript levels using the ΔΔCT method, displayed as mRNA fold change or Relative quantitation. The primers used for SYBR Green detection were designed by Quanta biosciences (Primer sequences are shown in Table S3).

miRNA expression was quantified in real-time SYBR Green qrt-PCR amplification reaction with the desired PerfeCta microRNA assay and the PerfeCta Universal PCR Primer following the manufacture's instruction (Quanta BioSciences, Inc.)

Western blot assay

For Western blot analyses, cells were lysed in RIPA buffer (50 mM Tris-HCl pH 8.0, 150 mM NaCl, 1% NP40, 0.1% SDS, 0.5% sodium deoxycholate, 1 mM PMSF, 25 mM MgCl2, supplemented with a phosphatase inhibitor cocktail). Protein concentration was determined by the Bradford protein assay (Bio-Rad Laboratories, Hercules, CA). Equivalent amounts of protein boiled in Laemmli sample buffer were electrophoresed on 10-12% SDS–polyacrylamide gels. PageRuler™ Prestained Protein Ladder (Fermentas, USA) was used to determine molecular weight. The gels were then electroblotted onto PVDF membranes. After blocking with 1% milk, membranes were incubated with specific primary anti-human antibodies against OCT4 (mouse anti-Oct4 (1:500, IgG2b; Santa Cruz Biotechnology, Inc., Santa Cruz, CA, USA)), Nanog (mouse anti-Nanog (1:2000; Millipore, New Jersey, USA, MABD 24clone 7F7.1)), CDH1 (mouse anti–E Cadherin (CDH1) (1:500; BD Pharmigen, CA, USA 610182 clone 36/E-Cadherin)), and beta-actin

(mouse anti beta-actin (1:15000; Sigma-Aldrich, St. Louis, MO, USA)). Finally, the relevant protein was visualized by staining with the appropriate secondary horseradish-peroxidase-labeled antibody for 1 h at room temperature. The reactions were detected by enhanced chemiluminescence assay-ECL (Biological Industries). The Signal was detected using a Bio-Rad image analyzer (Bio-Rad, Hercules, CA).

Cell proliferation and cell cycle analysis assays

Analysis of proliferation and cell cycle was carried out with bromodeoxyuridine (BrdU) labeling and the APC BrdU Flow Kit staining protocol (BD Pharmigen) according to the manufacturer's instructions. Briefly, $2x10^5$ NCCIT /shH19 and NCCIT/shLuc cells were plated onto 6 well plates and exposed for 3 days to Doxycycline hydrochloride (dox- 1microgram□/ml; Sigma Aldrich-Israel) in their growth medium. On day 3, the cells were pulsed with BrdU for 6 hours and then trypsinized and collected, washed with cold PBS, and fixed with 4% of paraformaldehyde (PFA) for 30 minutes. Fixed cells were resuspended in staining buffer containing 0.1% saponin (Sigma-Aldrich) and 30 microgram DNase and were incubated at 37^0C for 1 hour. Cells were then stained with anti-Brdu conjugated to APC (1:100 eBioscience, San Diego, CA USA) and analyzed for BrdU incorporation. The percentages of proliferating $BrdU^+$ GFP^+ cells were analyzed by FACS-calibur (Becton Dickinson Immunocytometry Systems), using CellQuest software. For cell cycle analysis, cells were stained with 10 microgram/ml 7-amino-actinomycin D (7-AAD; eBioscience) in the dark for 30 minutes. DNA content was detected using flow Cytometry analysis by FACScalibur (Becton Dickinson Immunocytometry Systems), using CellQuest software and according to the BrdU Flow Kit instruction manual (BD Pharmigen).

FACS analysis

hES and hEC cells were dissociated to a single-cell suspension. The cells were then washed with FACS buffer (PBS containing 1% BSA and 0.05% sodium azide-both from Sigma). The cells were incubated for 30 minutes with primary antibodies (Table S4). Control cells were stained with respective isotype control antibodies. Primary antibodies were detected using APC-labeled goat anti-mouse immunoglobulin M (1:100, Bioscience San Diego CA USA). PI was added to a final concentration of 4µg/ml for gating of viable cells. FACS analysis was performed using the FACSCalibur system (Becton Dickinson).

Annexin V assay for detection of apoptotic cells

The percentage of apoptotic cells was measured using Annexin V conjugated to APC apoptosis detection kit (BD Pharmigen) according to the manufacturer's protocol, in conjunction with the non-vital dye 7-aminoactinomysin (7-AAD). Briefly, harvested transduced and dox induced NCCIT cells ($1x10^6$) were washed with PBS, centrifuged and resuspended in Annexin buffer provided with the Kit. The final concentration used for Annexin was 0.5 microgram/microliter. Before analysis 7-AAD (5 µl/$1x10^6$cells) was added and cells were incubated for 5-10 minutes in the dark. Cells were analyzed by FACScalibur.

Immunofluorescence staining and confocal microscopy imaging

NCCIT/shLuc and NCCIT/shH19 cells were grown on poly-D-Lysine (10 mg/m; Sigma) and Laminin (4 mg/ml; Sigma) pre-coated glass coverslips for 8 days in the presence of dox (1microgram/ml). Cells were permeabilized in 0.5% Triton X-100/4% paraformaldehyde (PFA) and fixed in a 4% PFA/PBS-5% sucrose solution. Slides were blocked in PBS containing 10% normal donkey serum (Millipore). The cells were incubated with the following primary antibodies: anti-OCT-3/4 (mouse IgG, 1:100, Santa Cruz Biotechnology), anti-Nestin (rabbit 1:200 Millipore).

Primary antibodies were detected using RhodamineX-conjugated donkey anti-rabbit IgG and Cy5-conjugated donkey anti-Mouse IgG (Jackson Immunoresearch Labs, West Grove, PA, USA) or RhodamineX-conjugated donkey anti-rat IgG and Cy5 -conjugated donkey anti-Mouse IgG (Invitrogen). F-Actin staining was performed using the Alexafluor 488 phalloidin (1:200 Invitrogen). Mounting medium containing 4′6-diamidino-2-phenylindole (DAPI; Vector Burlingame, CA, USA) was used for nuclei counterstaining and imaging was performed by quadruple laser-assisted confocal microscopy (FluoView FV100, Olympus Tokio JApan), with a 60× objective.

Xenografts and formation of teratocarcinoma

Severe combined immune-deficient (SCID)/beige mice (C.B-17/ IcrHsd-SCID-bg) were maintained under defined flora conditions at the Hebrew University Pathogen-Free Animal Facility. All experiments were approved by the Animal Care Committee of the Hebrew University. Prior to transplantation transduced hEC cells (NCCIT/shLuc and NCCIT/shH19) were grown in the presence of dox (1μg/ml) for 3 days and then a sample of the cells was assayed by qPCR for H19 expression to check the efficiency of H19 knockdown. For transplantation, $1X10^6$ cells were resuspended in PBS to a total volume of 50 μl. The cell-mixture was combined with 50 μl undiluted cold Matrigel Basement membrane matrix (BD #354234, NJ, USA) immediately before transplantation. Pre-cooled pipettes, tips and syringes were used. The mixture of cells was injected subcutaneously into the flanks of the mice (100 μL containing 1×10^6 cells per mouse). The mice were divided into 2 groups: mice injected with (i) NCCIT/shLuc + dox (11 mice) and (ii) NCCIT/shH19 + dox (10 mice). The mice received dox (0.5 mg/ml) supplemented with 5% sucrose in their drinking water which was changed every other day. Once palpable, Tumors were measured in two dimensions using a vernier caliper, and tumor volume size (mm^3) was calculated with the formula V= $(\text{length}X\text{width}^2)/2$. Tumor growth was monitored for a period of 50 days until

tumors reached ethical restrictions. Teratocarcinoma tumor sections subjected to histological analysis by a certified pathologist.

Immunohistochemistry (IHC) analysis of xenograft tissue sections

The subcutaneous tumors formed in mice were fixed in 4% phosphate-buffered formalin and embedded in paraffin. Formalin-fixed, paraffin-embedded tissue samples (4-mm sections) were initially dewaxed, rehydrated and pretreated by antigen retrieval in 20 mM Citrate buffer (pH 6). Antibodies were hybridized in casblock buffer (Zymed Laboratories, San Francisco, CA, USA) for 30 minutes at room temperature and blocking of the endogenous peroxidase was performed with H202 (3%) (Merck,Darmstadt,Germany). Samples were then incubated overnight at 4°C in a humidified chamber with the specific primary anti-human antibodies against OCT4 (mouse anti-Oct4 (1:100, Santa Cruz Biotechnology)), Nanog (mouse anti-Nanog (1:100; Millipore)), CDH1 (mouse anti – E Cadherin (CDH1)(1:100; BD)) and SSEA1 (mouse anti-SSEA1 (1:100; Millipore)). Visualization was performed by using goat anti-mouse (Dako CA, USA). Chromogen substrate for HRP reaction was DAB plus (MA, USA) and sections were counterstained with H&E. For negative controls, primary antibody was omitted.

Table S1: siRNA duplexes:

Sense sequence	siRNA	Location
TAAGTCATTTGCACTGGTT	H19 RNA_1 (Presented data) (9)	Exon 5
CCAACATCAAAGACACCA	H19 RNA_3 (9)	Exon 5
CTTACGCTGAGTACTTCGATT	LUC RNA (Irrelevant control used in this study)	Exon 1

Table S2-Primers used for PCR

Gene	Primer sense	Primer anti-sense	Product size
**OCT4**	AGTGAGAGGCAACCTGGAGA	GTGAAGTGAGGGCTCCCATA	**273bp**
**Nanog**	CGAAGAATAGCAATGGTGTGACG	TTCCAAAGCAGCCTCCAAGTC	**328bp**
**Sox2**	GCCCTGCAGTACAACTCCAT	GGTAGTGCTGGGACATGTGA	**285bp**
**H19**	CAACCACTGCACTACCTGACTCA	CTGCTGTTCCGATGGTGTCTT	**302bp**
**GAPDH**	GACAACAGCCTCAAGATC	GTCCACCACTGACACGTT	**311bp**

Table S3-Primers used for qPCR (SYBR-Green Real-Time PCR)

Gene	primers sense (5' to 3')	Primer anti-sense (5' to 3')
H19 exon 4-5	CAACCACTGCACTACCTGACTCA	CTGCTGTTCCGATGGTGTCTT
H19 exon 1-2	GATCGGTGCCTCAGCGTT	TCATGTCCTGCTTGTCACGTC
hE-Cadherin (CDH1)	GCTTTGACGCCGAGAGCTA	CGGTGCAATCTTCAAAATTCACT
h-POU5F1(oct3/4)	TTGGAGACCTCTCAGCCTGAG	AGCTTCTCCTTCTCCAGCTTCA
h-Nanog	CTTCACCTATGCCTGTGATTTGTG	TGGAGGAAGGAAGAGGAGAGACA

h-N-Cadherin	TGCATCATCATCCTGCTTATCCT	GTTGTTTGGCCTGGCGTT
h-Vimentin	CGGCAGGTGGACCAGCTA	AAGCATCTCCTCCTGCAATTTCT
h-Keratin 18	AGACCGAGAACCGGAGGCT	TCCACAGTATTTGCGAAGATCTGA
h-Sox2	TCGAGATAAACATGGCAATCAA	AATTCAGCAAGAAGCCTCTCCTT
h-β-Actin	GAGCACAGAGCCTCGCCTT	CGATATCATCATCCATGGTGAGCT
Hsa-miR-675-5p		TGGTGCGGAGAGGGCCCACAGTG
Hsa-miR-675-3p		CTGTATGCCCTCACCGCTCA

Table S4- List of antibodies used in this study

Antibody	Manufacture/Cat. Number	Concentration/Dilution		
		Facs	IHC	WB
**Tra-1-60**	**Millipore mab 4360**	**1:100**		
**Tra-1-81**	**Millipore mab 4381**	**1:100**		
**SSEA1**	**Biolegend 125601 (MC 480)**	**1:100**	**1:100**	
**BrdU APC**	**eBioscience 17-5071 (BU20A)**	**1:100**		
**CDH1**	**BD 610181 (36/E-Cadherin)**		**1:100**	**1:500**
**OCT4**	**Santa Cruz(cat sc 5279)**	**1:100**	**1:100**	**1:500**
**Nanog**	**Millipore MABD24 (7F7.1)**	**1:100**	**1:100**	**1:2000**
**Nestin**	**Millipore AB5922**		**1:200**	

Supplementary References

1. Ben-Dor, I., Itsykson, P., Goldenberg, D., Galun, E.,Reubinoff, B. E. Lentiviral vectors harboring a dual-gene system allow high and homogeneous transgene expression in selected polyclonal human embryonic stem cells. Mol Ther, *14:* 255-267, 2006.
2. van de Wetering, M., Oving, I., Muncan, V., Pon Fong, M. T., Brantjes, H., van Leenen, D., et al. Specific inhibition of gene expression using a stably integrated, inducible small-interfering-RNA vector. EMBO Rep, *4:* 609-615, 2003.
3. Zufferey, R., Dull, T., Mandel, R. J., Bukovsky, A., Quiroz, D., Naldini, L., et al. Self-inactivating lentivirus vector for safe and efficient in vivo gene delivery. J Virol, *72:* 9873-9880, 1998.
4. Gropp, M., Itsykson, P., Singer, O., Ben-Hur, T., Reinhartz, E., Galun, E., et al. Stable genetic modification of human embryonic stem cells by lentiviral vectors. Mol Ther, *7:* 281-287, 2003.
5. Gropp, M.,Reubinoff, B. Lentiviral vector-mediated gene delivery into human embryonic stem cells. Methods Enzymol, *420:* 64-81, 2006.
6. Naldini, L., Blomer, U., Gallay, P., Ory, D., Mulligan, R., Gage, F. H., et al. In vivo gene delivery and stable transduction of nondividing cells by a lentiviral vector. Science, *272:* 263-267, 1996.
7. Dull, T., Zufferey, R., Kelly, M., Mandel, R. J., Nguyen, M., Trono, D., et al. A third-generation lentivirus vector with a conditional packaging system. J Virol, *72:* 8463-8471, 1998.
8. Gropp, M.,Reubinoff, B. E. Lentiviral-RNA-interference system mediating homogenous and monitored level of gene silencing in human embryonic stem cells. Cloning Stem Cells, *9:* 339-345, 2007.
9. Matouk, I. J., DeGroot, N., Mezan, S., Ayesh, S., Abu-lail, R., Hochberg, A., et al. The H19 non-coding RNA is essential for human tumor growth. PLoS One, *2:* e845, 2007.

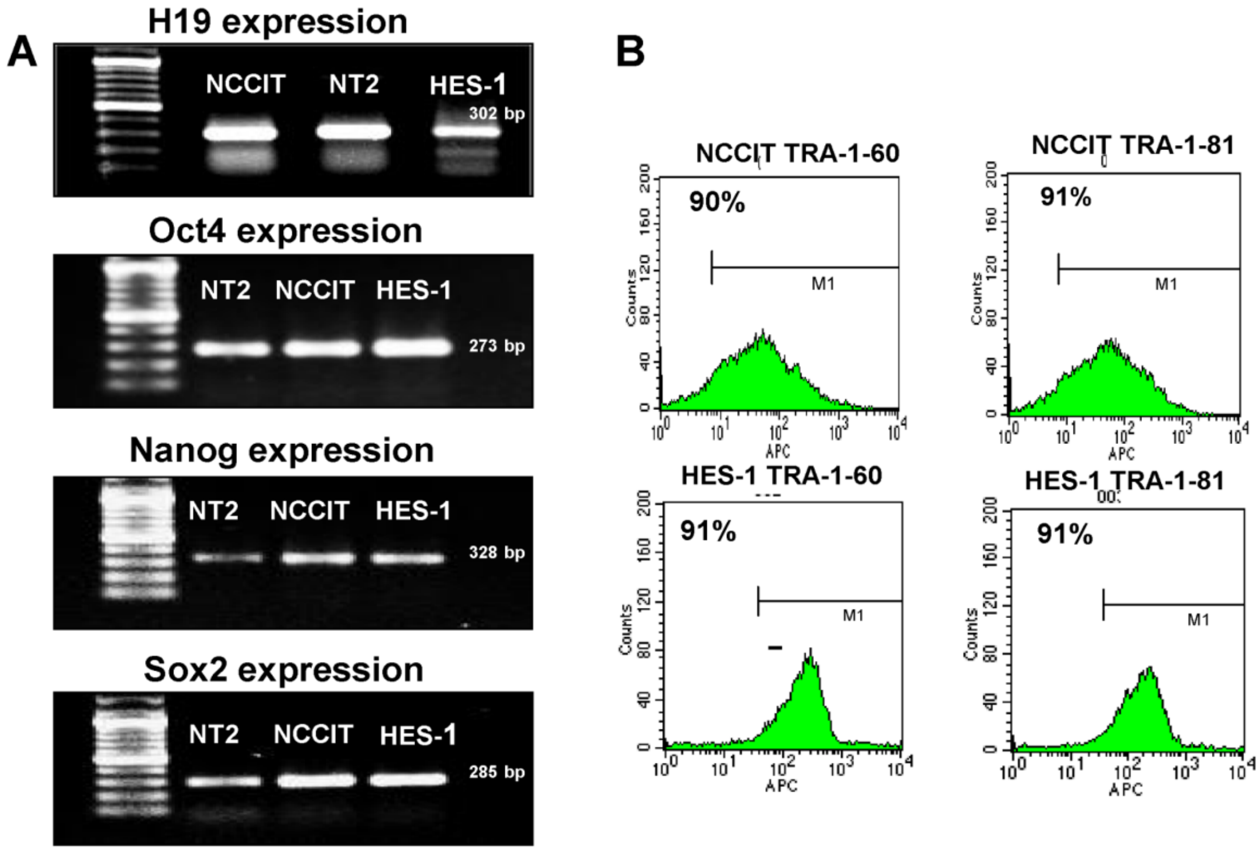

Figure S1. Human ES and EC cells express H19 and pluripotency markers.

(A) RT-PCR analysis showing an expression of H19, Oct4, Nanog and Sox2 in NCCIT, NT2 and HES-1 cells. (B) FACS analysis showing the expression of the cell surface antigens TRA-1-60 and TRA-1-81 in NCCIT and HES-1 cells.

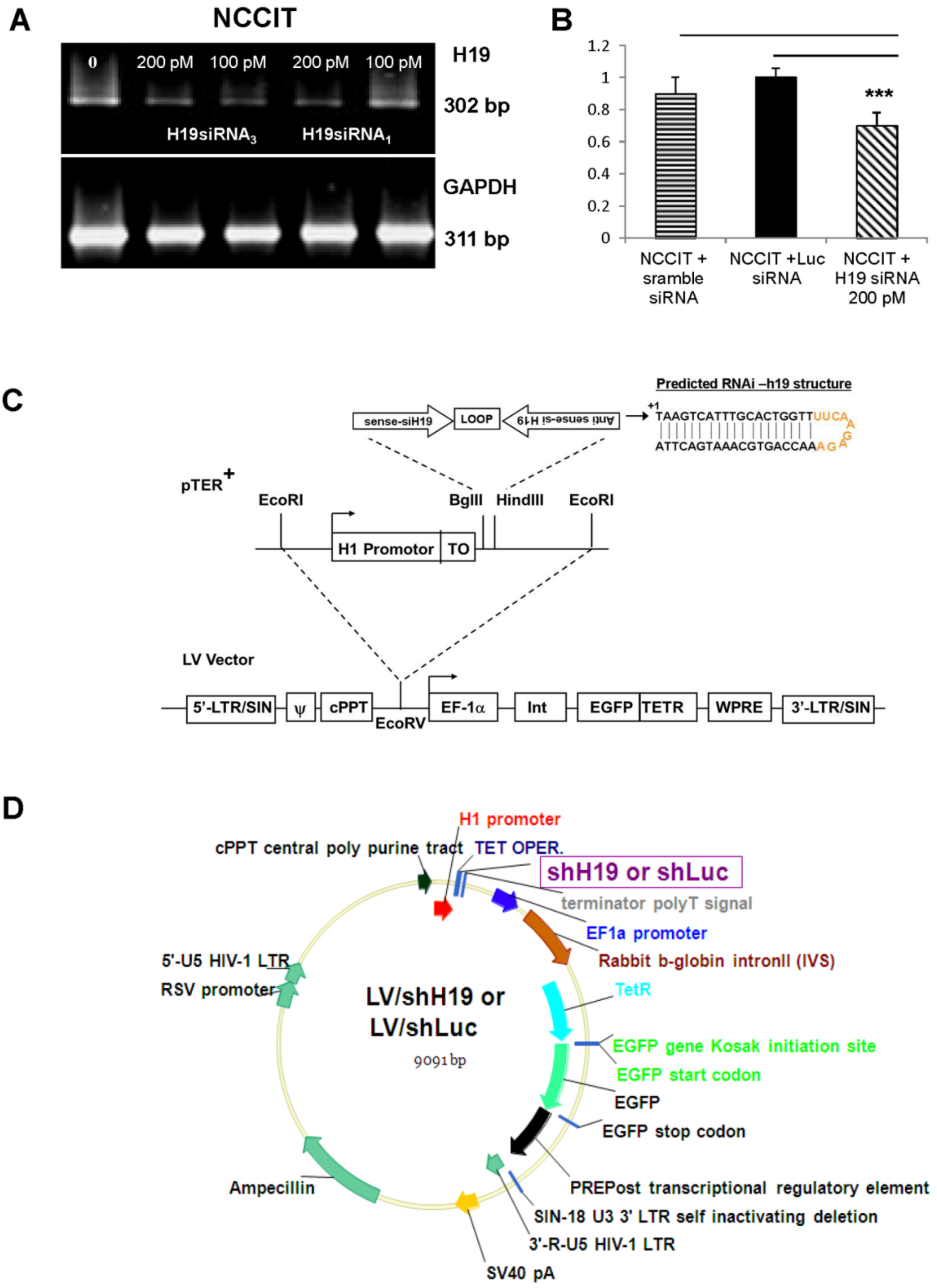

Figure S2. **Design of a lentivirus vector for the expression of H19 and Luc specific siRNA**.

(A) NCCIT cells were transiently transfected with H19 siRNA $_1$ and H19 $siRNA_3$ both at concentrations of 200pM and 100pM. The RT PCR results show efficient down regulation of H19s with both siRNA. (B) Real time PCR of transiently transfected NCCIT cells with two controls: Luc siRNA, H19 siRNA and scramble siRNA. The results show that both controls were similar and H19 was efficiently down-regulated. (C) The siRNA (siH19) was directionally inserted into the Bgl II and HindIII sites of pTER+ a modified pTER Plasmid. The complete shH19/shLuc expression cassette was excised from the pTER+ as an EcoRI fragment (Blunt ends) and inserted into the EcoRV of the lentiviral vector pLV (dotted line). LTR indicates long terminal repeat; ψ, psi packaging signal; cPPT, central polypurine tract; EF1-α, human elongation factor alpha; EGFP, enhanced green fluorescent protein fused to TETR, tet repressor-Tetracycline-controlled transcriptional activation; WPRE, posttranscriptional cis-acting regulatory element of the woodchuck hepatitis virus; and LTR/SIN, self-inactivating 3′ long terminal repeat. (D) The final modified lentiviral vector (LV/sH19/shLuc).

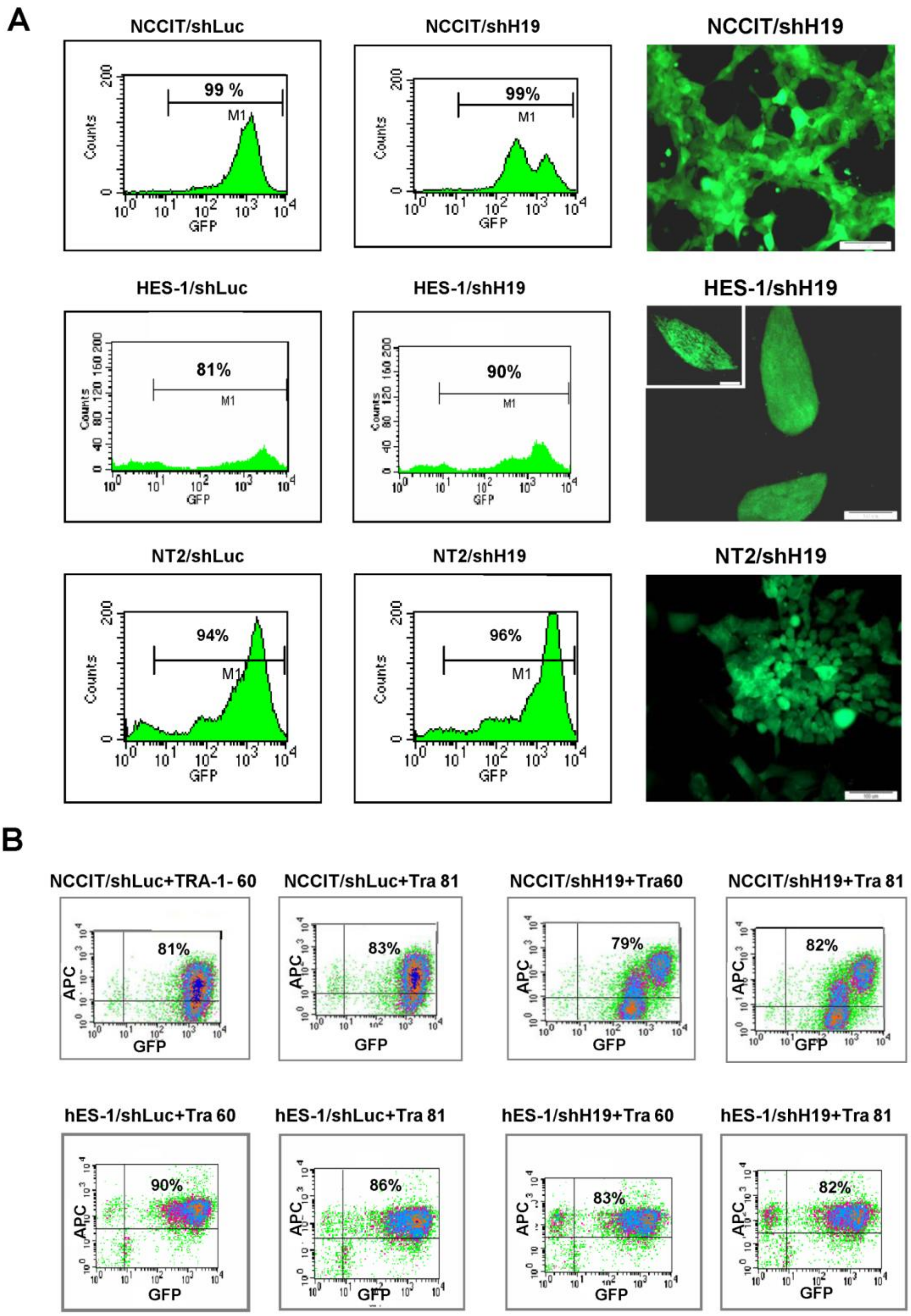

Figure S3. Transduction of hES and hEC cells with lentiviral vectors harboring a tet-inducible H19-shRNA or a control luciferase (Luc)-shRNA.

(A) Efficient and stable transduction of the HES-1, NCCIT and NT2 cells with LV/shH19 or the negative control LV/shLuc. (B) FACS analysis of pluripotency surface antigen markers TRA-1-60 and Tra-1-81 showing that the transduction slightly reduced the percentages of these markers.
